# Intravenous perfluorocarbons for prevention of ventilator-associated ARDS

**DOI:** 10.1186/cc12039

**Published:** 2013-03-19

**Authors:** A Scultetus, A Haque, F Arnaud, G McNamee, C Auker, R McCarron, P McKay, R Mahon

**Affiliations:** 1 Naval Medical Research Center, Silver Spring, MD, USA; 2 Uniformed Services University of the Health Sciences, Bethesda, MD, USA

## Introduction

Emulsified perfluorocarbons (PFC) are synthetic hydrocarbons that can carry 50 times more oxygen than human plasma. Their properties may be advantageous in applications requiring preservation of tissue viability in oxygen-deprived states [1,2], making them a potential candidate for combat and civilian prehospital resuscitation. Our hypothesis is that an intravenous dose of PFC increases vital organ tissue oxygenation, improves survival after hemorrhagic shock (HS) and may reduce or prevent the development of ventilator-associated ARDS. Here we report data from the first part (HS only) of a multiphase swine study to study the benefits of PFC in treating HS and preventing ARDS. This initial study was designed to ensure safe use of PFC in traumatized animals.

## Methods

Anesthetized Yorkshire swine were hemorrhaged 55% of their estimated blood volume (Time 0 (T0)) over 15 minutes. At T15 minutes, pigs received a bolus of the PFC Oxycyte or 10% hydroxyethyl starch (HES). At T60 animals underwent continuous hemorrhage (0.5 cm^3^/kg/minute) until death. Time to death and physiological parameters were primary endpoints.

## Results

Average survival time after onset of second hemorrhage was 60 minutes in PFC-treated animals versus 65 minutes in the HES group. Mean arterial pressure (MAP) was similar between T0 and T60, thereafter PFC-treated animals had lower MAP, mean pulmonary artery pressure (MPAP), heart rate and cardiac output (*P >*0.05).

## Conclusion

There was no significant difference in survival time, MAP and MPAP in the PFC group compared with HES control. These data suggest that it is safe to administer in this HS model. Regulatory approval of hemoglobin-based oxygen carriers has been halted due to possible side effects related to vasoconstriction. In this model, and with this class of oxygen-carrying drugs (PFCs), we did not observe evidence of vasoconstriction. Using PFCs did not result in a survival advantage here; however, there was also no observation of adverse events. Based on these data we will continue to the next phase of this project and test PFC in the prevention of ARDS alone, and in combination with HS.
